# Supplementary material for: Riparian vegetation reduces coastal turbidity
Source: Commun Sustain. 2026 Feb 10;1(1):29. doi: 10.1038/s44458-025-00031-5 (PMC12885955; doi:10.1038/s44458-025-00031-5)
Supplement: Supplementary file 1 — Supplementary Information [file 44458_2025_31_MOESM1_ESM.pdf]

Riparian vegetation reduces coastal turbidity

Supplementary Information

Supplementary Note 1: Results details

Other notable covariate impacts

NDTI at the river mouth directly up-current was also a particularly strong and consistent predictor (e.g., estimate: 0.405 for the model in Fig. 2A), highlighting spatial autocorrelation in turbidity patterns. Given the likely similarity in land use between neighboring river systems, this up-current variable may absorb some of the explanatory power that might otherwise be attributed to local treatment effects, making our estimates of land use treatment effects more conservative.

Sensitivity analysis results

Sensitivity analysis, testing the robustness of our estimates to potential omitted variable bias, suggests that the statistically significant treatment effects of the IPW models with random effects were robust to some unobserved confounding. The IPW model with random effects with riparian natural vegetation as the treatment had a robustness value of 0.192, indicating that any unmeasured confounder that explained more than 19.2% of the residual variance in both the treatment and the outcome could nullify the observed effect of riparian natural vegetation on NDTI 100m off the coast. The robustness value calibrated to the 5% significant level was 0.102, indicating that an unobserved confounder explaining 10.2% of the residual variance would make the observed relationship statistically non-significant. The IPW model with riparian pasture as the treatment had a smaller robustness value (0.154) and robustness at the 5% significant level (0.059). These robustness estimates are likely underestimated, due to the parameterization[1] and because random effects were not included in the models since *Sensemakr* does not work on linear mixed effects models, reducing the estimated effect sizes of natural and pasture in the models tested for robustness.

The direction of the effect (negative) of riparian natural vegetation is robust to confounding up to five times as strong as the observed confounder presence of gravel roads, although the effect size gets slightly reduced from the original estimate in each case (Supplementary Figure 7A). At the 5% significance level, the null hypothesis of zero effect would still be rejected given unobserved confounders up to twice as strong as presence of gravel roads (Supplementary Figure 7B). The direction of the effect is robust to confounding at least three times as strong as the observed confounder watershed natural vegetation (Supplementary Figure 7C), and the null hypothesis would still be rejected given unobserved confounders twice as strong as watershed natural vegetation (Supplementary Figure 7D). These are conservative estimates, as it is highly unlikely to have as strong of an unobserved confounder as strong as the confounding relationship between watershed natural vegetation and riparian natural vegetation.

Common support results

Inverse probability of treatment weighting tended to increase common support between the treated and untreated rivers. In comparing the covariate balance for models with full riparian natural land as the treatment, rivers were considered "treated" if their full riparian zones were more than 50% forested for ease of visualization (Supplementary Figure 8). Many covariates had little common support in the treated and untreated groups before weighing. The continuous baseline variables and the tails of the distributions differed less in the weighted sample than in the unweighted sample.

NDTI validation

Remotely sensed NDTI statistically significantly predicted the *in situ* NTU measurements (Supplementary Figure 9). Pearson and Spearman correlation indicated a moderate to strong monotonic relationship between NDTI and NTU. NDTI was an even stronger predictor of log-transformed NTU (Supplementary Figure 9B).

There are several factors that may have reduced the ability of NDTI to predict NTU. Bias and error in the field NTU measurements may have arisen from factors such as time of day, weather, and sampling variability. For instance, Field NTU varied based on the time of day it was collected, with highest values recorded between 9am and 1pm (Supplementary Figure 10). When time of day of NTU data collection was included as a predictor in the models, NDTI was still a significant predictor of NTU. NDTI values were derived from the Landsat 8 scene temporally closest to each field sampling event, but image acquisition was sometimes up to a month apart due to cloud cover and the satellite's revisit schedule. This temporal mismatch, particularly during the rainy season, introduced uncertainty. Furthermore, cloud cover limited satellite observations during the wet season, reducing the temporal resolution and lowering confidence in seasonal NDTI averages compared to dry-season and annual estimates. There might also be a non-linear relationship between NDTI and NTU.

Supplementary Note 2: Comparing causal inference modelling approaches

IPW model assumptions

IPW models require assumptions, some of which are unique to IPW and many of which are held by all causal inference modeling approaches. Assumptions for IPW include a) no unmeasured confounding, b) positivity, c) no interference, d) consistency, e) no noncompliance, and f) no misspecifications of the model[2–5]. Here, we consider all six of these assumptions in turn in relation to our analysis.

IPW models rely heavily on the assumption that there are no unobserved confounders[2,5] Given that this is a complex socioecological system, it is possible that there is unobserved confounding. However, the sensitivity analysis suggests that effects of the impacts of riparian natural vegetation and pasture on turbidity would still be statistically significant even if there are unobserved confounders that explain up to 19.2% and 15.4% of the residual variance, respectively. For instance, if there were unobserved confounders up to five times as strong as the presence of gravel roads or three times as strong as watershed natural vegetation, riparian natural vegetation would still have a statistically significant negative impact on gulf turbidity (Supplementary Figure 7). These are conservative estimates, and it is very unlikely that we missed a confounder as strong as watershed natural vegetation or gravel roads. Second, every riparian zone and every watershed has a nonzero probability of having any treatment land use or roads, so the positivity assumption holds[2].

No interference and consistency are two components of the stable unit treatment value assumption (SUTVA)[6]. Interference could be caused by spillover effects or deforestation leakage. However, Brumberg et al. (2024)[7] found increases in forest cover both inside and outside of protected areas, suggesting that deforestation leakage is unlikely in the Osa. No interference also assumes that the turbidity at one river mouth is only dependent on that river's treatment land use and not on the treatment land use of another river[4]. Gulf currents may lead to interference, because land use in a given river may affect gulf turbidity both at the mouth of that river and at the mouth of down-current rivers. We included the gulf turbidity from the most proximal up-current river as a covariate in the models to control for this interference. The consistency assumption requires that there are not multiple versions of the treatment[4,5]. In this system, there is variation within each of the treatments. For example, an area that is considered natural vegetation could be mature forest, newly regenerating secondary forest, or mangroves; a pasture could be abandoned or actively grazing cattle; and gravel roads vary drastically in their use frequency. Each of these multiple versions of the natural vegetation, pasture, and gravel roads may have different impacts on turbidity. While detailed data on the pasture and road use is not available, models were run separately for forest mature forest, secondary forest, and mangrove and wetland, and they had similar treatment effects. The large clustered robust standard error (RSE) of estimates for natural, plantation, and exposed land uses could be in part due to multiple versions of the treatment (Supplementary Figure 6).

Noncompliance occurs when a unit's actual condition differs from the treatment condition[4]. In this case, it would occur if the land use proportion or presence of roads does not accurately reflect *in situ* conditions, which is possible since these treatment data were extracted from remotely sensed products. It is unlikely that there is much land use non-compliance, as the LULC maps have a 90.44% accuracy[7]. The most common misclassifications were palm and pasture misclassified as forest[7]. Consequently, there may be some non-compliance in the natural vegetation class which includes forest, potentially leading to a conservative estimate of the effect of natural vegetation on reducing turbidity. IPW assumes that the propensity score model is properly specified, although the use of propensity scores makes estimated treatment effects less sensitive to misspecified models[3]. Covariate balance and common support–overlap between covariate values and treatment values or weights–are two aspects of a properly specified model[2–5]. When the continuous riparian land use treatments were divided into categorical treatment groups, the continuous baseline variables and the tails of the distributions differed less in the weighted sample than in the unweighted sample (Supplementary Figure 8).

Comparing results of modelling approaches

We compared traditional linear models to four causal inference methods: IPW with and without random effects, two-way fixed effects, group mean covariate, and group mean centered models. The consistent direction of effects—negative for riparian natural vegetation and positive for riparian pasture—across all models (Figure 6) reinforces the robustness of these associations.

Across all models, the estimated effect of riparian natural vegetation on turbidity was consistently negative and statistically significant (Figure 6A), reinforcing evidence for a plausible causal relationship. For riparian pasture, estimates were consistently positive, but only four models—mixed-effects, IPW with random effects, group mean covariate, and group mean centered—found statistically significant effects (Figure 6B), indicating the association is weaker and more sensitive to modeling assumptions.

The IPW models with clustered robust standard errors (RSE) but no random effects produced slightly different results than those with random effects and standard SEs (Supplementary Figure 6). The model without random effects found that riparian natural vegetation significantly reduces NDTI (estimate: -0.0636, 95% CI: -0.120 to -0.008), but the effect was about half the size of the model with random effects, and the confidence interval was wider. Similarly, the estimate for riparian pasture decreased and became non-significant ($p=0.084$) when using clustered RSE, due to larger standard errors. In contrast, results for riparian gravel road presence were nearly identical across model types, likely because these models were only run on 2019 data, so random effects were not included. Interestingly, the clustered RSE for watershed gravel road presence was smaller than the standard SE, resulting in a significant positive effect (estimate: 0.075, 95% CI: 0.031 to 0.118). No other models showed statistically significant effects.

The IPW model with random effects yielded a substantially larger treatment effect for riparian natural vegetation than the naïve model, suggesting that key confounders (e.g., other land uses, slope, soil type) were appropriately accounted for. In contrast, riparian pasture only reached statistical significance in models that incorporated either random effects or addressed watershed-level confounding (group mean covariate or centered models), indicating that its relationship with turbidity may be more context-dependent or subject to residual confounding. Effect sizes were broadly similar between IPW models with random effects and those using clustered robust standard errors (RSEs), but statistical significance differed. Clustered RSEs, which adjust for heteroskedasticity and within-cluster correlation[8], were generally more conservative, leading to loss of significance for riparian pasture. However, for watershed gravel road presence, clustered RSEs were actually smaller than standard errors, yielding a statistically significant effect. This highlights how different error structures can affect inference, even when point estimates are similar.

Group Mean Covariate and Group Mean Centered models produced the largest effect sizes for riparian pasture. By accounting for unobserved watershed-level heterogeneity, these models reduce potential bias from omitted variables such as geomorphology or regional land management[6]. The similarity in effect sizes between these models and IPW suggests that IPW assumptions were not substantially violated. Two-Way Fixed Effects models control for time-invariant and time-varying unobserved confounding at the river level[6]. These models did not find a significant effect of pasture on turbidity, which may reflect either unresolved spatiotemporal confounding or limited within-site temporal variability in pasture cover. It is possible that fixed effects absorbed much of the relevant variation, reducing the ability to detect an effect.

In sum, similar effect sizes across modeling strategies support the robustness of the riparian natural vegetation findings. Differences in statistical significance for pasture highlight the importance of considering model-specific assumptions, error structures, and the role of both observed and unobserved confounding. Our estimates of local land use treatment effects are likely underestimates because the NDTI at the nearest up-current river mouth variable included in all models was strongly statistically significant. Given the likely similarity in land use between neighboring watersheds, this variable may absorb explanatory power for the treatment land use.

Supplementary Note 3: Coral reef case study

To demonstrate how future research might integrate coral reef health or other marine ecosystem field data into their analyses, we compiled all the known data on coral reef health in the Golfo Dulce and compared it to NDTI. We intend this information to be illustrative: to build the narrative of the canopy-to-coral relationship, and to help inform future work; no statistical analysis was conducted, and we do not endeavor to assess causality.

Methods

Coral reef health data, defined as live coral proportion, were collected *in situ* at six coral reefs by marine biologists at the Centro de Investigación en Ciencias del Mar y Limnología (CIMAR) at the University of Costa Rica[9–11]. Each coral reef was sampled two to five times between 1988 and 2017 for a total of 23 observations. The belt-quadrat method was used, and the areas of each quadrat containing live coral, dead coral, and other substrates (rubble and/or sand) were recorded following the methods in Cortés (1990). The percentage of each reef composed of live coral was then estimated, and these values ranged from 0.05 to 76.7% over the study period. The El Niño in 2015-2016 is considered one of the most severe ever worldwide, causing a 75% decline in live coral in the Golfo Dulce[9]. To better disentangle the impacts of land use on live coral coverage, we did not include *in situ* samples from 2017 in the analysis, as their live coral coverage was driven by the El Niño impacts.

NDTI was extracted during the five years prior to each field reef sampling following the methods described in the "Gulf turbidity and depth" subsection of the Methods in the main text. Mean NDTI was calculated for the three months, one year, and five years prior to reef sampling. The number of extreme turbidity events during the five years prior to sampling was estimated. Extreme turbidity events were defined as NDTI observations within the five years prior to sampling with values greater than the seventy-fifth percentile of all observations for all reefs (NDTI > -0.3612). Analysis of the relationship between coral reef health and turbidity was exploratory and purely visual due to the extremely small sample size and variation in the dates of coral reef health data collection.

Results

Given the small sample size of six coral areas and the complexity of coral reef systems, we did not conduct statistical modeling on the CVV data, so we cannot fully isolate the influence of land use and turbidity from other ecological drivers (ex. El Niño/La Niña, temperature, wave power)[9,12]. This preliminary dataset shows a potential negative correlation between live coral cover (CVV) and mean NDTI, particularly when averaged over the five years preceding coral measurements (Supplementary Figure 11).

Punta Islotes, one of only two reefs with longitudinal coral cover data, offers a compelling case study. Located down current of the Río Esquinas—a major watershed heavily deforested for banana cultivation in the 1940s—the reef exhibited severe degradation by 1987, with live coral cover reduced to just 1.7% of its area and the substrate dominated by algae and fine sediment[10,11,13]. Riparian vegetation regenerated in subsequent decades (from 53% to 72%, while pasture and exposed soil declined sharply). These changes were paralleled by reductions in turbidity at both the river mouth and Punta Islotes, with NDTI values decreasing from -0.14 to -0.18 (100m offshore) and from 0.01 to -0.13 (800m offshore). This coincided with a marked recovery: live coral cover increased from 1.7% in 1987 to 9.9% by 1997 and reached 16.9% by the mid-2010s. While causality cannot be conclusively established, the temporal alignment of increased vegetation, reduced turbidity, and coral recovery suggests a plausible link. These patterns align with local ecological history and broader tropical reef research, indicating that riparian land use—particularly deforestation and subsequent regeneration—can influence coral reef health via turbidity.

It is important to note that coral reef health in the Golfo Dulce is shaped by multiple interacting factors. For instance, Hurricane Caesar in 1996 buried much of the Sándalo reef, likely obscuring any signal from land use changes[9,11]. Other pressures—including illegal fishing, salinity and temperature fluctuations, and reef-specific assemblages of bioeroders—further complicate attribution[11,14]. Species-specific sensitivity to turbidity and the limitations of live coral cover as a proxy for sub-lethal impacts on growth or structure add further complexity[10,11,15]. Future studies could expand temporal and spatial sampling to enable multivariate analyses that control for confounding biotic and abiotic drivers. Beyond turbidity, land use may influence reefs via nutrient enrichment, chemical pollutants, and altered sediment characteristics. Understanding species-specific sensitivities to these stressors is particularly critical in under-studied regions like Pacific Costa Rica[14].

Supplementary Figures

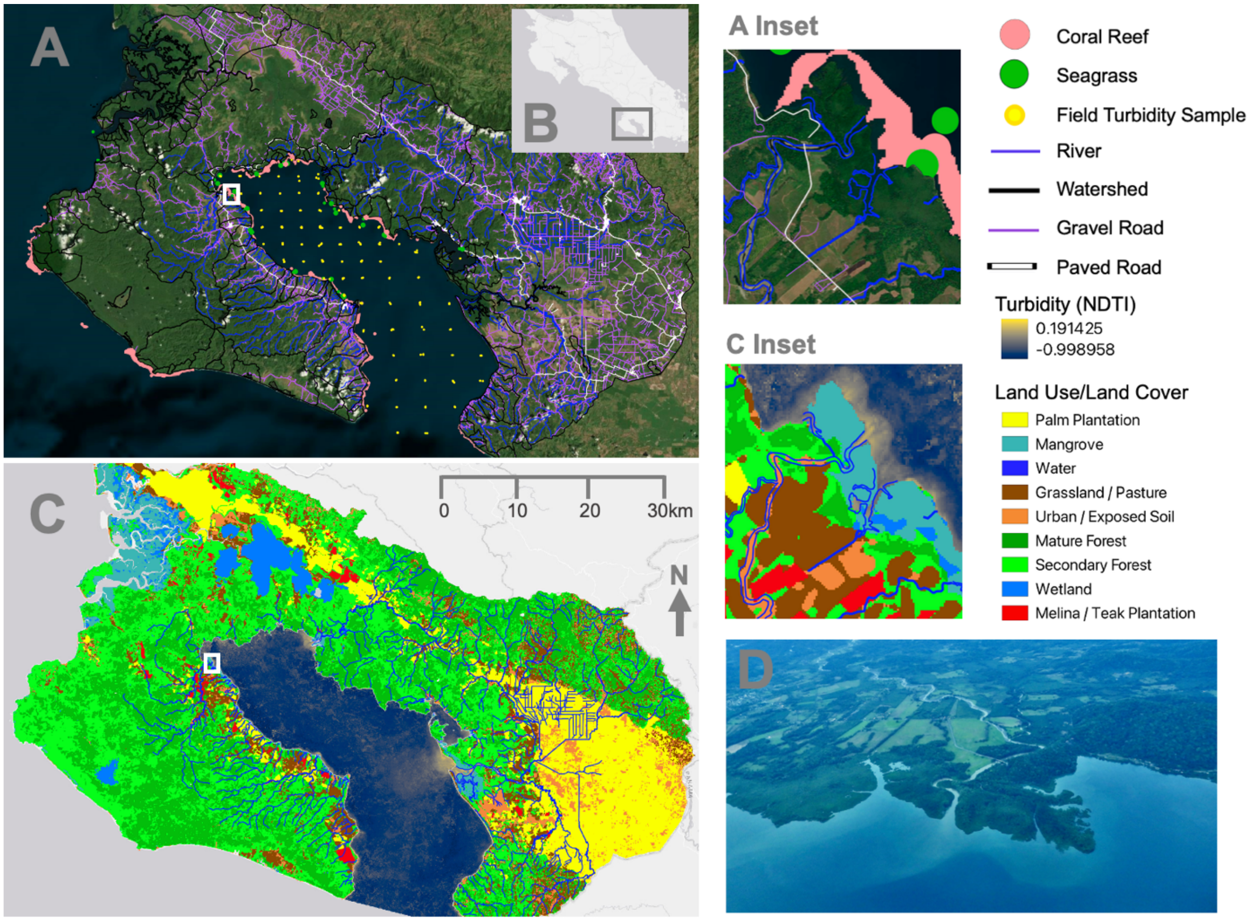

Supplementary Figure 1. Study area map with inset maps to visualize relationships between key variables. A: Study area and focal variables. Coral reefs (coral circles), seagrass beds (green circles), and field turbidity measurements (yellow). All rivers that flow into the Golfo Dulce (dark blue lines), watersheds (black lines), gravel roads (purple lines), and paved roads (white lines). B: Location of the study area in panel A within Costa Rica. C: Land use / land cover and turbidity (Normalized Difference Turbidity Index: NDTI) from 2019, along with all rivers that flow into the Golfo Dulce (dark blue). Yellow indicates higher turbidity, and darker blue indicates lower turbidity. Inset maps showing the Rincón-Riyito River mouth (outlined in white rectangles in panels A and C) are provided to help illustrate the relationship between land use, turbidity, coral reefs, and seagrass beds. Panel "A Inset" shows the same features as panel A, while panel "C Inset" same features at panel C. D: Aerial photograph of Rincón-Riyito River flowing into Golfo Dulce.

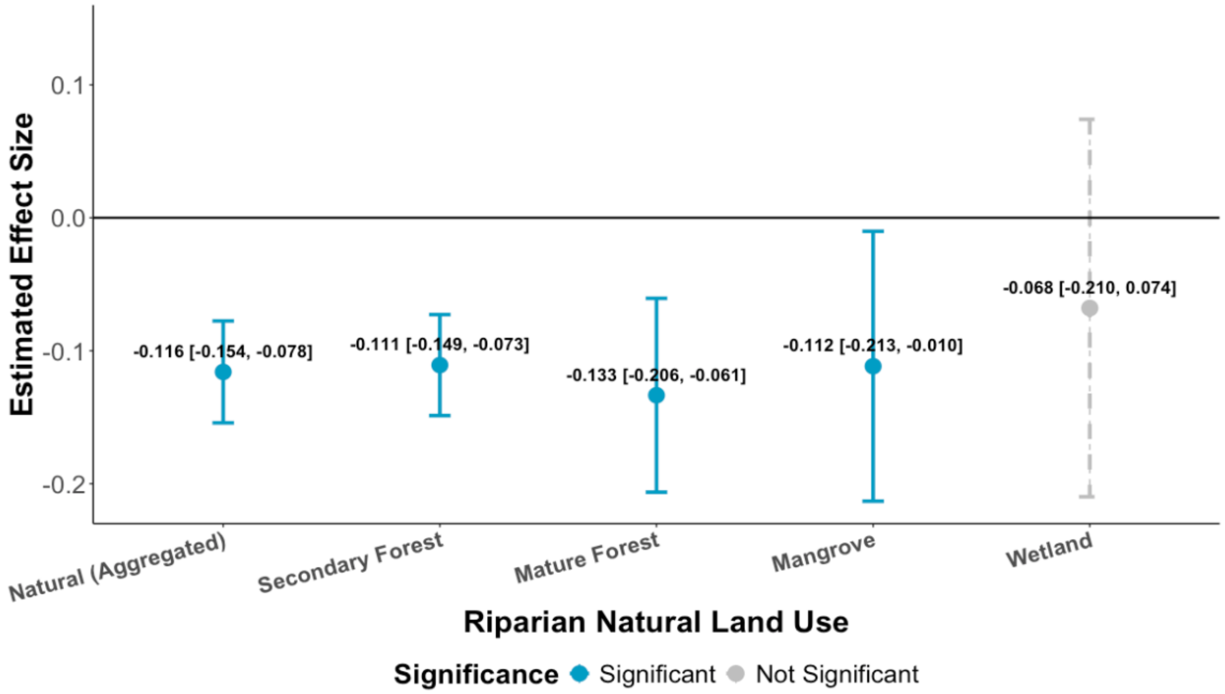

Supplementary Figure 2. Estimated effects of riparian natural vegetation types on coastal turbidity. Estimates from inverse probability of treatment weighting (IPW) models with random effects of the impact of the proportion of each natural vegetation type on annual turbidity 100m offshore. Numbers indicate the estimate with the 95% confidence interval (CI) in brackets. Bars illustrate 95% CIs. Blue indicates a p-value of the estimate less than 0.05, and gray indicates a p-value greater than 0.05.

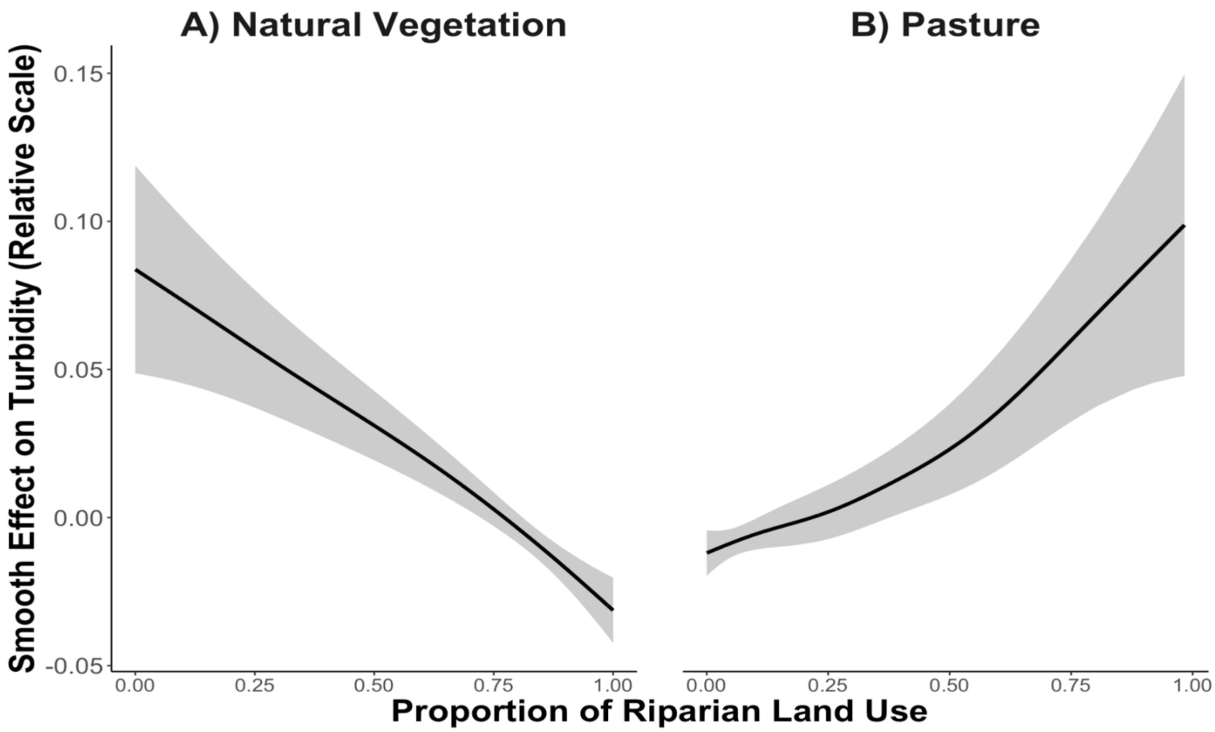

Supplementary Figure 3. Smooth effects from IPW generalized additive mixed models (GAMMs) estimating the effect of the proportion of A) riparian natural vegetation and B) riparian pasture on predicted coastal turbidity (NDTI) 100m offshore in Costa Rica. The y-axis shows the smooth contribution of each land use, holding other covariates constant. Shaded areas represent 95% confidence intervals.

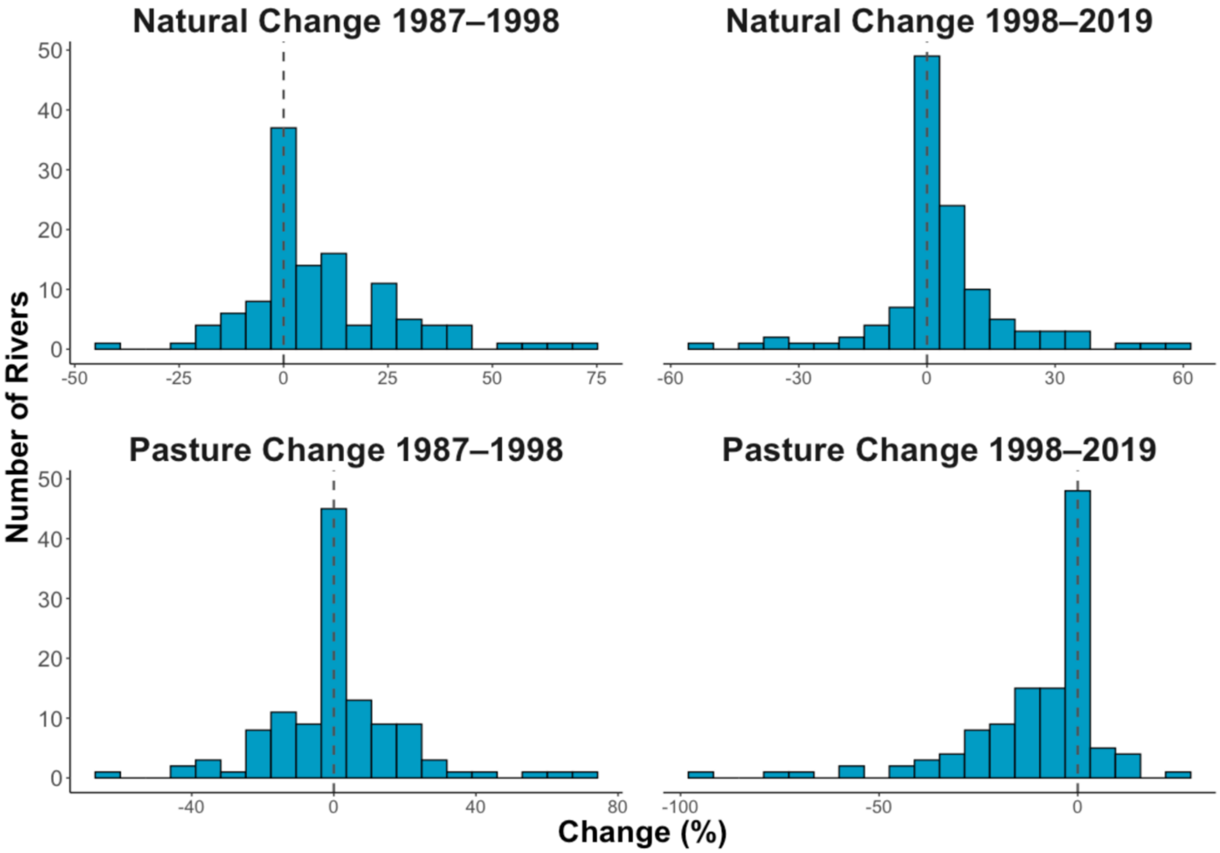

Supplementary Figure 4. Change in percentage of riparian natural vegetation (top panels) and pasture (bottom panels) between 1987-1998 and 1998-2019.

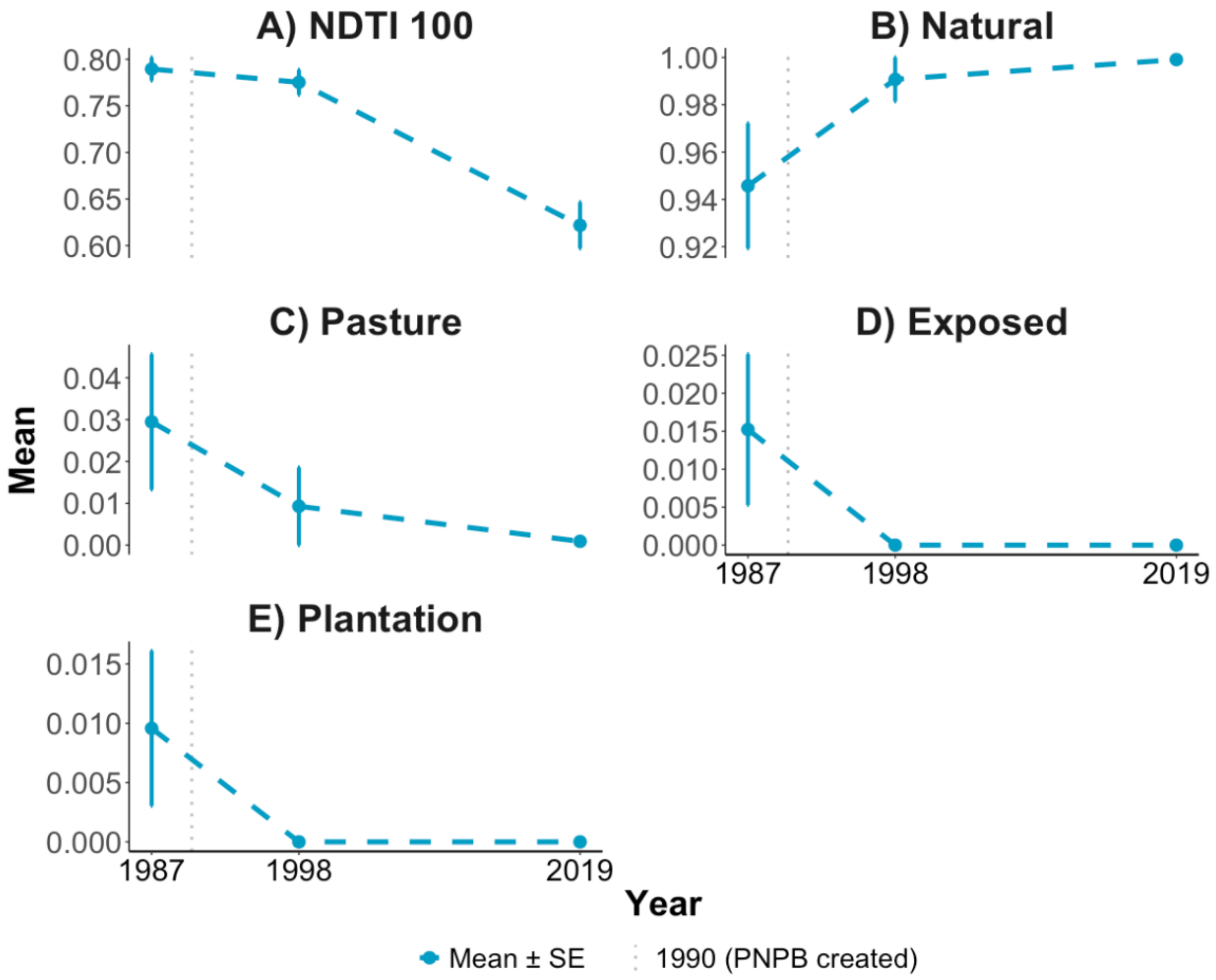

Supplementary Figure 5. Piedras Blancas National Park (PBNP) trends from 1987 to 2019 in A) turbidity (NDTI) 100m offshore, and the proportion of riparian zones covered by B) natural vegetation, C) pasture, D) exposed land, and E) plantation vegetation along the 12 rivers that flow from PBNP into the Golfo Dulce. Estimates represent mean values and standard error across rivers. The dotted vertical line marks the year 1990, when PBNP was established[16].

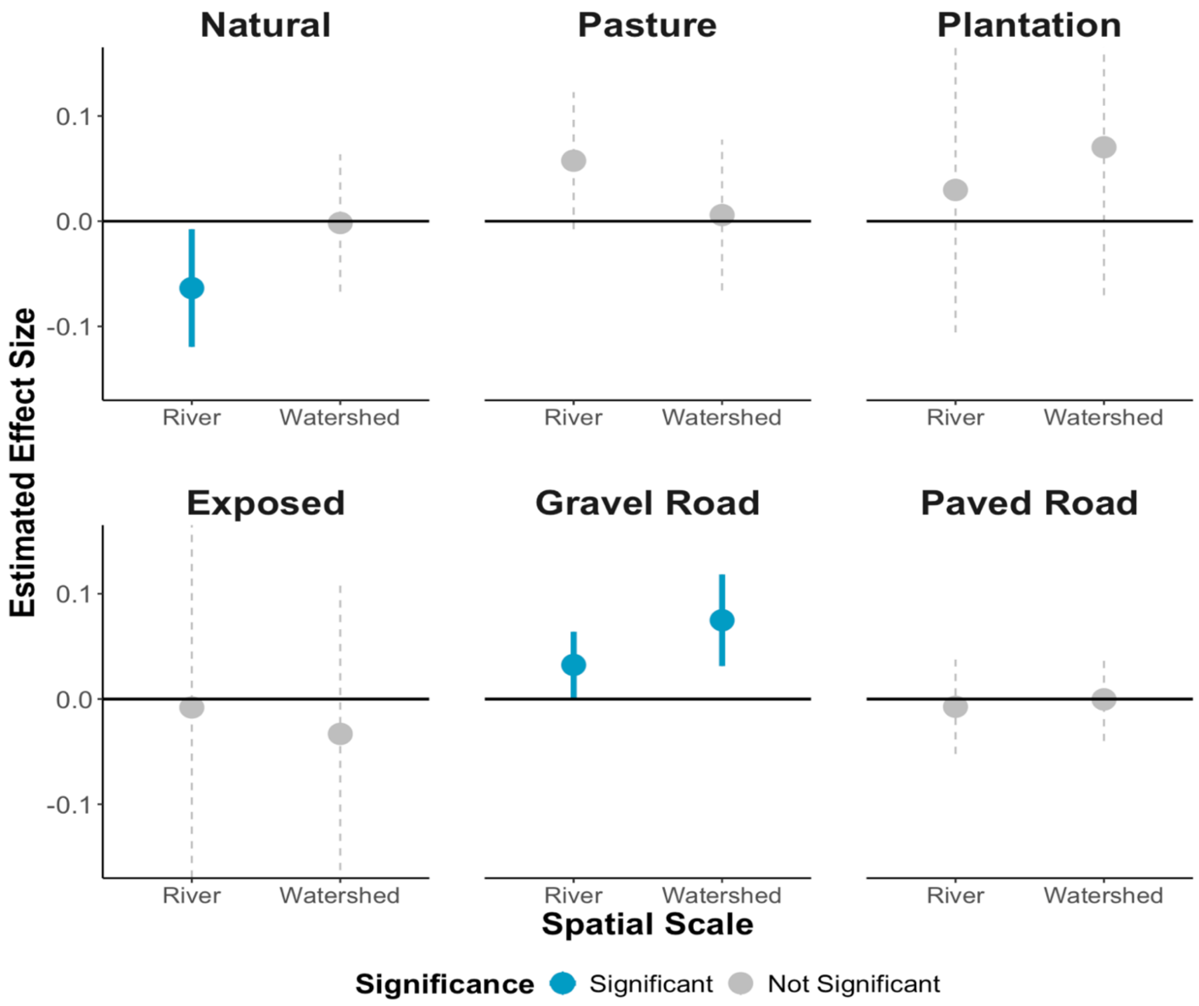

Supplementary Figure 6. Estimates from IPW models with clustered robust standard errors (without random effects) of the impact of the proportion of A) natural vegetation, B) pasture, C) plantation, D) exposed land, and the presence of E) gravel road, and F) paved road in riparian zones and in watersheds on average annual turbidity 100m offshore. Bars indicate clustered robust standard errors. Blue indicates a p-value of the estimate less than 0.05, and gray indicates a p-value greater than 0.05.

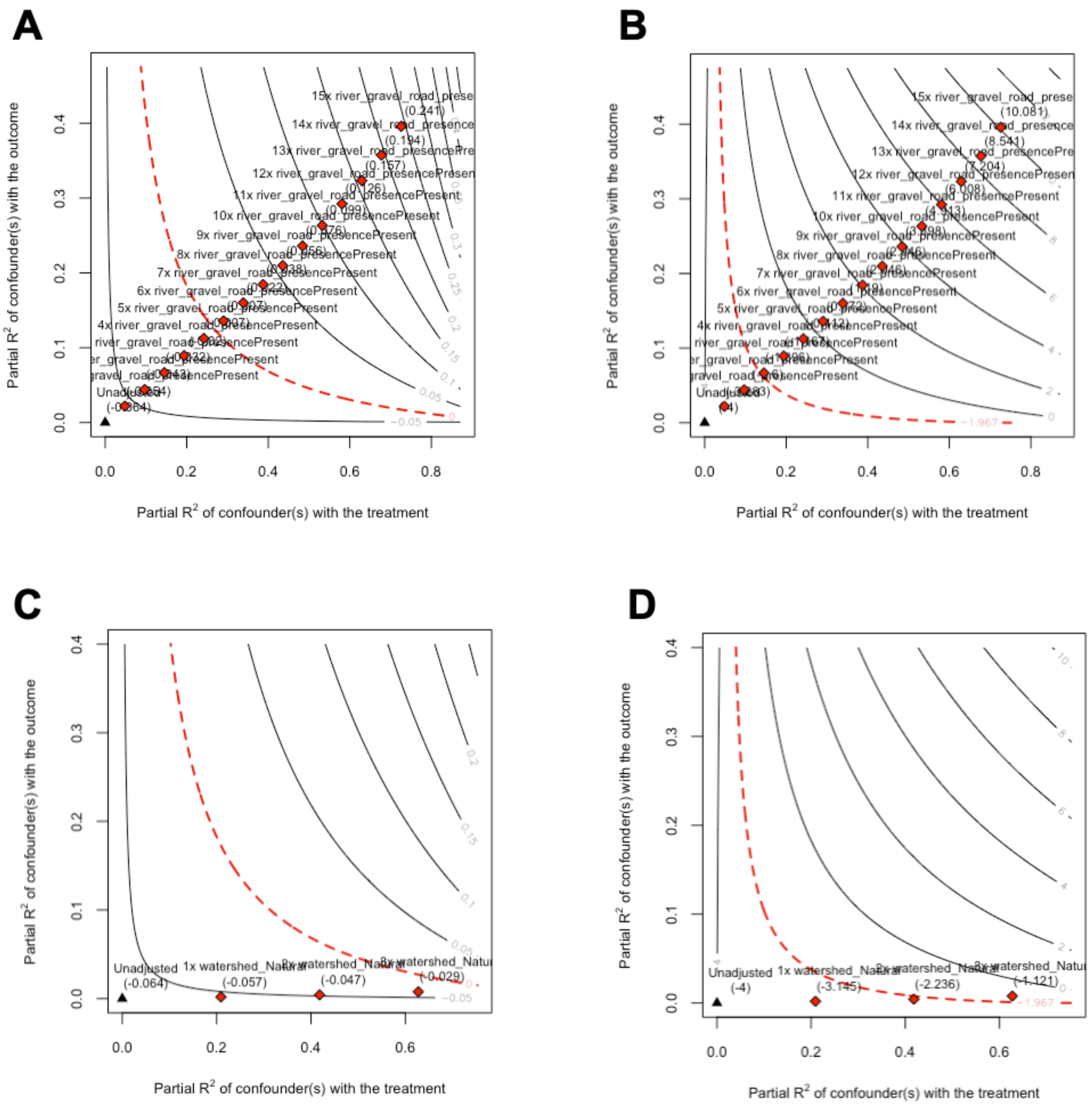

Supplementary Figure 7. Sensitivity contour plots of point estimates and t-values for the IPW model of riparian natural vegetation to an unobserved confounder with a similar strength to the presence of gravel roads (A-B) or to watershed natural vegetation (C-D). Panels A and C are the contour plots for the point estimate, and panels B and D are for the 5% significance level. The X-axes show the hypothetical residual share of variation of the treatment (riparian natural vegetation) that unobserved confounding could explain, and the Y-axis indicates the hypothetical share of variation of the response (annual NDTI 100m offshore) that unobserved confounding could explain.

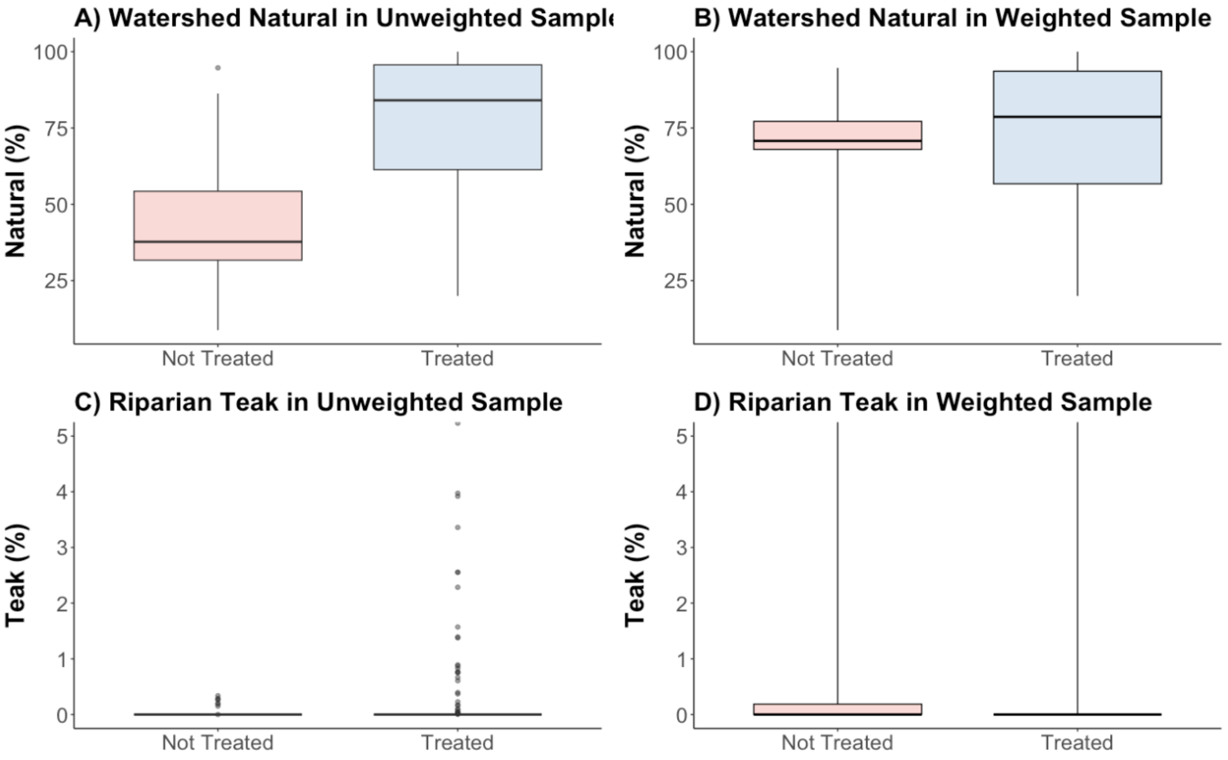

Supplementary Figure 8. Distribution of watershed natural vegetation (A-B) and riparian teak (C-D) in treated and untreated rivers in the unweighted (A,C) and weighted (B,D) sample based on weights for riparian natural vegetation as the treatment. Weights are from the model of the impact of riparian natural vegetation. “Treated” rivers have more than 50% natural vegetation in their riparian zones.

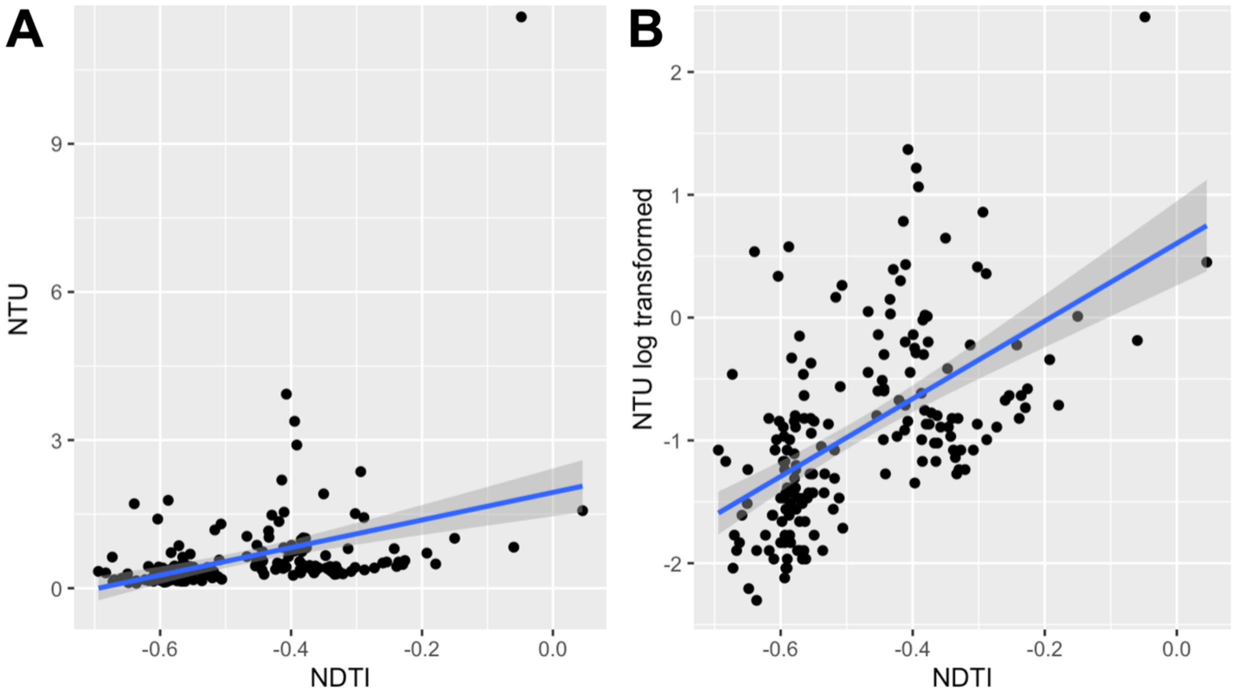

Supplementary Figure 9. Relationships between remotely sensed NDTI from the Landsat imagery most proximal to the field data collection and A) field NTU measurement (slope=2.7941), and B) log transformed field NTU measurement (slope=3.1642).

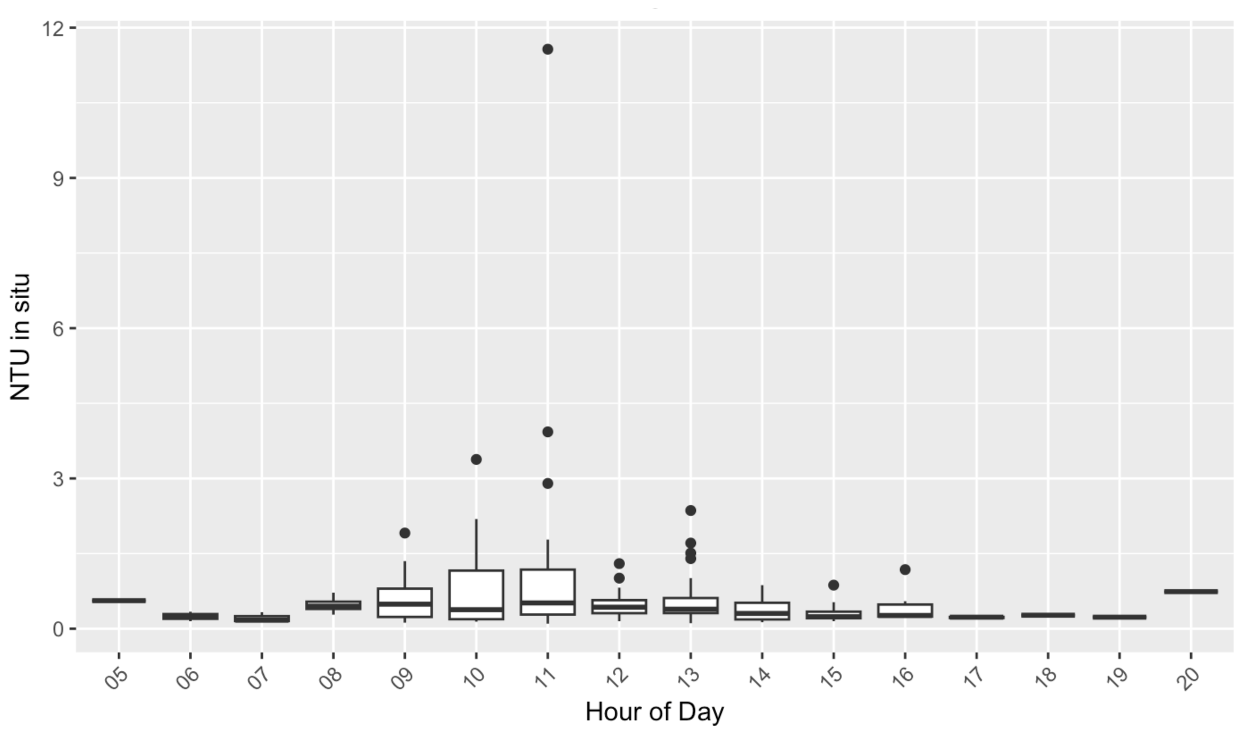

Supplementary Figure 10. Boxplot of field turbidity values (NTU, Nephelometric Turbidity Unit) in the Golfo Dulce based on the hour of data collection.

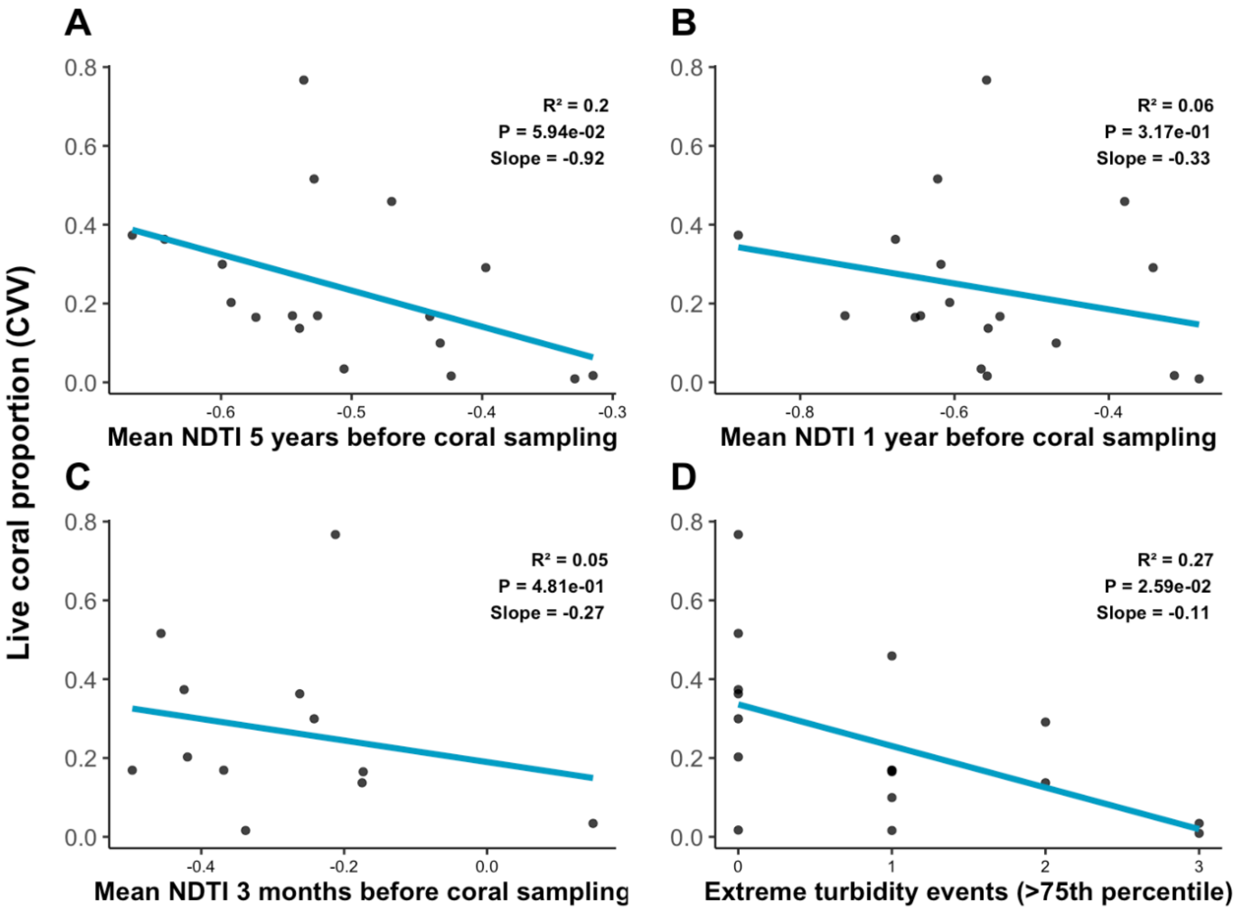

Supplementary Figure 11. Relationship between live coral proportion (CVV) from small *in situ* dataset and mean turbidity during three time periods prior to *in situ* coral reef sampling: A) five years, B) one year, C) three months, and D) relationship between CVV and number of extreme turbidity events during the five years prior to sampling. Extreme turbidity events were defined as NDTI observations within the five years prior to sampling with values greater than the seventy-fifth percentile of all observations for all reefs (NDTI > -0.3612).

Supplementary Table S1. Estimates for the effect size of land use at two spatial scales on gulf turbidity 100m offshore. Values correspond to Figure 2. Blue rows indicate statistically-significant estimates ($p<0.05$).

**Treatment land use**	**Scale**	**Estimate**	**Standard error**	**Low bounds of 95% confidence interval**	**High bounds of 95% confidence interval**	**P-value**	**Significant?**
Pasture	Watershed	0.0247	0.0360	-0.0461	0.0955	0.4930	No
Pasture	River	0.0828	0.0195	0.0446	0.1211	0.0000	Yes
Natural	Watershed	-0.0195	0.0353	-0.0891	0.0501	0.5815	No
Natural	River	-0.1159	0.0195	-0.1543	-0.0776	0.0000	Yes
Exposed	Watershed	-0.0781	0.0545	-0.1854	0.0291	0.1528	No
Exposed	River	-0.0464	0.0524	-0.1495	0.0567	0.3768	No
Plantation	Watershed	0.0272	0.0586	-0.0882	0.1425	0.6434	No
Plantation	River	0.0307	0.0407	-0.0494	0.1108	0.4511	No
Gravel Road	Watershed	0.0749	0.0381	-0.0006	0.1504	0.0519	No
Gravel Road	River	0.0324	0.0154	0.0018	0.0630	0.0381	Yes
Paved Road	Watershed	-0.0003	0.0184	-0.0369	0.0362	0.9850	No
Paved Road	River	-0.0074	0.0266	-0.0601	0.0453	0.7824	Yes

Supplementary Table S2. Estimates for the effect of riparian land use in the dry season, wet season, and year average on turbidity in the gulf 25-800m offshore. Values correspond to Figure 3. Blue rows indicate statistically-significant estimates (p<0.05). Table can also be accessed in editable format in the Harvard Dataverse repository.[17]

Treatment land use	Time period	Distance offshore (m)	Estimate	Standard error	P-value	Significant?
Natural	Dry Season	25	-0.0854	0.0216	0.0001	Yes
Natural	Dry Season	50	-0.0963	0.0228	0.0000	Yes
Natural	Dry Season	75	-0.0895	0.0247	0.0003	Yes
Natural	Dry Season	100	-0.0811	0.0252	0.0014	Yes
Natural	Dry Season	125	-0.0855	0.0255	0.0009	Yes
Natural	Dry Season	150	-0.0897	0.0254	0.0005	Yes
Natural	Dry Season	175	-0.0833	0.0259	0.0015	Yes
Natural	Dry Season	200	-0.0612	0.0257	0.0177	Yes
Natural	Dry Season	300	-0.0324	0.0237	0.1722	No
Natural	Dry Season	400	-0.0178	0.0250	0.4757	No
Natural	Dry Season	500	-0.0491	0.0235	0.0376	Yes
Natural	Dry Season	600	-0.0279	0.0224	0.2146	No
Natural	Dry Season	700	-0.0498	0.0198	0.0125	Yes
Natural	Dry Season	800	-0.0497	0.0196	0.0116	Yes
Natural	Rainy Season	25	-0.0343	0.0221	0.1228	No
Natural	Rainy Season	50	-0.0482	0.0227	0.0352	Yes
Natural	Rainy Season	75	-0.0606	0.0266	0.0237	Yes
Natural	Rainy Season	100	-0.0469	0.0277	0.0911	No
Natural	Rainy Season	125	-0.0478	0.0273	0.0817	No
Natural	Rainy Season	150	-0.0286	0.0275	0.3003	No
Natural	Rainy Season	175	-0.0238	0.0289	0.4106	No
Natural	Rainy Season	200	-0.0144	0.0289	0.6178	No
Natural	Rainy Season	300	-0.0128	0.0306	0.6760	No
Natural	Rainy Season	400	-0.0613	0.0363	0.0924	No
Natural	Rainy Season	500	-0.0331	0.0361	0.3600	No
Natural	Rainy Season	600	-0.0175	0.0386	0.6506	No
Natural	Rainy Season	700	-0.0331	0.0388	0.3945	No
Natural	Rainy Season	800	-0.0372	0.0420	0.3767	No
Natural	Full Year	25	-0.1006	0.0168	0.0000	Yes
Natural	Full Year	50	-0.1000	0.0173	0.0000	Yes
Natural	Full Year	75	-0.0941	0.0188	0.0000	Yes
Natural	Full Year	100	-0.1159	0.0195	0.0000	Yes
Natural	Full Year	125	-0.1217	0.0197	0.0000	Yes
Natural	Full Year	150	-0.1237	0.0195	0.0000	Yes
Natural	Full Year	175	-0.1095	0.0201	0.0000	Yes

Natural	Full Year	200	-0.0944	0.0203	0.0000	Yes
Natural	Full Year	300	-0.0915	0.0207	0.0000	Yes
Natural	Full Year	400	-0.1296	0.0242	0.0000	Yes
Natural	Full Year	500	-0.1078	0.0240	0.0000	Yes
Natural	Full Year	600	-0.1203	0.0236	0.0000	Yes
Natural	Full Year	700	-0.1044	0.0234	0.0000	Yes
Natural	Full Year	800	-0.1158	0.0241	0.0000	Yes
Pasture	Dry Season	25	0.0637	0.0197	0.0014	Yes
Pasture	Dry Season	50	0.0600	0.0212	0.0050	Yes
Pasture	Dry Season	75	0.0384	0.0230	0.0962	No
Pasture	Dry Season	100	0.0348	0.0232	0.1349	No
Pasture	Dry Season	125	0.0406	0.0230	0.0780	No
Pasture	Dry Season	150	0.0437	0.0230	0.0581	No
Pasture	Dry Season	175	0.0293	0.0232	0.2062	No
Pasture	Dry Season	200	0.0163	0.0231	0.4794	No
Pasture	Dry Season	300	-0.0025	0.0219	0.9102	No
Pasture	Dry Season	400	-0.0105	0.0254	0.6787	No
Pasture	Dry Season	500	0.0101	0.0241	0.6752	No
Pasture	Dry Season	600	0.0095	0.0226	0.6749	No
Pasture	Dry Season	700	0.0328	0.0200	0.1017	No
Pasture	Dry Season	800	0.0304	0.0189	0.1082	No
Pasture	Rainy Season	25	0.0340	0.0229	0.1384	No
Pasture	Rainy Season	50	0.0377	0.0233	0.1075	No
Pasture	Rainy Season	75	0.0429	0.0259	0.0981	No
Pasture	Rainy Season	100	0.0160	0.0266	0.5468	No
Pasture	Rainy Season	125	0.0157	0.0259	0.5460	No
Pasture	Rainy Season	150	-0.0004	0.0258	0.9882	No
Pasture	Rainy Season	175	-0.0011	0.0271	0.9683	No
Pasture	Rainy Season	200	-0.0065	0.0273	0.8127	No
Pasture	Rainy Season	300	-0.0130	0.0284	0.6479	No
Pasture	Rainy Season	400	0.0380	0.0318	0.2325	No
Pasture	Rainy Season	500	0.0026	0.0323	0.9355	No
Pasture	Rainy Season	600	0.0001	0.0369	0.9970	No
Pasture	Rainy Season	700	0.0069	0.0375	0.8532	No
Pasture	Rainy Season	800	0.0031	0.0394	0.9374	No
Pasture	Full Year	25	0.0826	0.0164	0.0000	Yes
Pasture	Full Year	50	0.0734	0.0171	0.0000	Yes
Pasture	Full Year	75	0.0563	0.0190	0.0032	Yes
Pasture	Full Year	100	0.0828	0.0195	0.0000	Yes

Pasture	Full Year	125	0.0824	0.0191	0.0000	Yes
Pasture	Full Year	150	0.0789	0.0187	0.0000	Yes
Pasture	Full Year	175	0.0786	0.0191	0.0000	Yes
Pasture	Full Year	200	0.0677	0.0192	0.0005	Yes
Pasture	Full Year	300	0.0612	0.0191	0.0015	Yes
Pasture	Full Year	400	0.0755	0.0237	0.0016	Yes
Pasture	Full Year	500	0.0560	0.0240	0.0201	Yes
Pasture	Full Year	600	0.0981	0.0231	0.0000	Yes
Pasture	Full Year	700	0.0853	0.0216	0.0001	Yes
Pasture	Full Year	800	0.0927	0.0216	0.0000	Yes
Gravel Road	Dry Season	25	0.0116	0.0175	0.5093	No
Gravel Road	Dry Season	50	0.0212	0.0189	0.2658	No
Gravel Road	Dry Season	75	0.0450	0.0207	0.0319	Yes
Gravel Road	Dry Season	100	0.0590	0.0192	0.0027	Yes
Gravel Road	Dry Season	125	0.0609	0.0193	0.0021	Yes
Gravel Road	Dry Season	150	0.0567	0.0189	0.0034	Yes
Gravel Road	Dry Season	175	0.0662	0.0187	0.0006	Yes
Gravel Road	Dry Season	200	0.0771	0.0177	0.0000	Yes
Gravel Road	Dry Season	300	0.0411	0.0156	0.0097	Yes
Gravel Road	Dry Season	400	0.0016	0.0153	0.9168	Yes
Gravel Road	Dry Season	500	-0.0103	0.0130	0.4271	No
Gravel Road	Dry Season	600	0.0030	0.0114	0.7939	No
Gravel Road	Dry Season	700	-0.0085	0.0108	0.4355	No
Gravel Road	Dry Season	800	-0.0068	0.0108	0.5270	No
Gravel Road	Rainy Season	25	0.0199	0.0174	0.2561	No
Gravel Road	Rainy Season	50	0.0171	0.0181	0.3475	No
Gravel Road	Rainy Season	75	0.0214	0.0191	0.2646	No
Gravel Road	Rainy Season	100	0.0267	0.0190	0.1645	No
Gravel Road	Rainy Season	125	0.0232	0.0193	0.2305	No
Gravel Road	Rainy Season	150	0.0183	0.0191	0.3407	No
Gravel Road	Rainy Season	175	0.0228	0.0201	0.2586	No
Gravel Road	Rainy Season	200	0.0286	0.0202	0.1590	No
Gravel Road	Rainy Season	300	0.0106	0.0226	0.6409	No
Gravel Road	Rainy Season	400	-0.0233	0.0238	0.3292	No
Gravel Road	Rainy Season	500	-0.0280	0.0237	0.2391	No
Gravel Road	Rainy Season	600	-0.0037	0.0247	0.8816	No
Gravel Road	Rainy Season	700	0.0038	0.0271	0.8886	No
Gravel Road	Rainy Season	800	-0.0170	0.0334	0.6117	No
Gravel Road	Full Year	25	0.0196	0.0140	0.1643	No

Gravel Road	Full Year	50	0.0230	0.0144	0.1123	No
Gravel Road	Full Year	75	0.0300	0.0159	0.0625	No
Gravel Road	Full Year	100	0.0324	0.0154	0.0381	Yes
Gravel Road	Full Year	125	0.0325	0.0159	0.0432	Yes
Gravel Road	Full Year	150	0.0283	0.0158	0.0754	No
Gravel Road	Full Year	175	0.0369	0.0159	0.0226	Yes
Gravel Road	Full Year	200	0.0439	0.0157	0.0062	Yes
Gravel Road	Full Year	300	0.0247	0.0162	0.1293	No
Gravel Road	Full Year	400	-0.0214	0.0187	0.2535	No
Gravel Road	Full Year	500	-0.0321	0.0183	0.0830	No
Gravel Road	Full Year	600	-0.0006	0.0181	0.9757	No
Gravel Road	Full Year	700	-0.0028	0.0185	0.8817	No
Gravel Road	Full Year	800	-0.0138	0.0204	0.5003	No

Supplementary Table S3. Estimates for interactions between each significant treatment from Figure 2 and river variables. Significant models are illustrated in Figure 5. Blue rows indicate statistically-significant estimates ($p<0.05$).

Treatment	River variable	Estimate of interaction term	P-value	Significant?
Pasture	Discharge	-1.34	0.0000	Yes
Pasture	Length	-0.17	0.0001	Yes
Pasture	Slope	0.20	0.3542	No
Gravel	Discharge	0.42	0.1569	No
Gravel	Length	0.29	0.6217	No
Gravel	Slope	0.17	0.2615	No
Natural	Discharge	0.88	0.0067	Yes
Natural	Length	0.07	0.2200	No
Natural	Slope	-0.49	0.0024	Yes

Supplementary References

1. Cinelli, C. & Hazlett, C. An introduction to sensitivity analysis using sensemakr. (2024).
2. Ramsey, D. S. L., Forsyth, D. M., Wright, E., McKay, M. & Westbrooke, I. Using propensity scores for causal inference in ecology: Options, considerations, and a case study. *Methods in Ecology and Evolution* **10**, 320–331 (2019).
3. Austin, P. C. & Stuart, E. A. Moving towards best practice when using inverse probability of treatment weighting (IPTW) using the propensity score to estimate causal treatment effects in observational studies. *Statistics in Medicine* **34**, 3661–3679 (2015).
4. Kimmel, K., Dee, L. E., Avolio, M. L. & Ferraro, P. J. Causal assumptions and causal inference in ecological experiments. *Trends in Ecology & Evolution* **36**, 1141–1152 (2021).
5. Chesnaye, N. C. *et al.* An introduction to inverse probability of treatment weighting in observational research. *Clinical Kidney Journal* **15**, 14–20 (2022).
6. Byrnes, J. E. K. & Dee, L. E. Causal inference with observational data and unobserved confounding variables. 2024.02.26.582072 Preprint at https://doi.org/10.1101/2024.02.26.582072 (2024).
7. Brumberg, H. *et al.* Increasing Forest Cover and Connectivity Both Inside and Outside of Protected Areas in Southwestern Costa Rica. *Remote Sensing* **16**, 1088 (2024).
8. Stock, J. H. & Watson, M. W. Heteroskedasticity-Robust Standard Errors for Fixed Effects Panel Data Regression. *Econometrica* **76**, 155–174 (2008).
9. Alvarado, J. J. *et al.* Impact of El Niño 2015-2016 on the coral reefs of the Pacific of Costa Rica: the potential role of marine protection. *Revista de Biología Tropical* **68**, S271-282 (2020).
10. Cortés, J. The coral reefs of Golfo Dulce, Costa Rica: distribution and community structure. *Atoll Research Bulletin* **344**, 1–37 (1990).
11. Fonseca, A., Dean, H. K. & Cortés, J. Non-colonial coral macro-borers as indicators of coral reef status in the south Pacific of Costa Rica. *Revista de Biología Tropical* **54**, 101–115 (2006).

12. Delevaux, J. M. S. *et al.* Place-based management can reduce human impacts on coral reefs in a changing climate. *Ecological Applications* **29**, e01891 (2019).

13. Cortés, J., Macintyre, I. & Glynn, P. Holocene growth history of an eastern Pacific fringing reef, Punta Islotes, Costa Rica. *Coral Reefs* **13**, 65–73 (1994).

14. Cortés, J. & Jiménez, C. Corals and coral reefs of the Pacific of Costa Rica: history, research and status. in *Latin American Coral Reefs* (ed. Cortés, J.) 361–385 (Elsevier Science, Amsterdam, 2003). doi:10.1016/B978-044451388-5/50017-5.

15. Cortés & Risk, M. J. A reef under siltation stress: Cahuita, Costa Rica. *Bulletin of Marine Science* **36**, 339–356 (1985).

16. SINAC. Parque Nacional Piedras Blancas: Historia. https://www.sinac.go.cr/ES/ac/acosa/pnpb/Paginas/default.aspx.

17. Brumberg, H. *et al.* Riparian vegetation reduces coastal turbidity. Harvard Dataverse https://doi.org/10.7910/DVN/IBCSCB (2025).
