# Supplementary material for: Riparian vegetation reduces coastal turbidity
Source: Commun Sustain. 2026 Feb 10;1(1):29. doi: 10.1038/s44458-025-00031-5 (PMC12885955; doi:10.1038/s44458-025-00031-5)
Supplement: Supplementary file 2 — Reporting Summary [file 44458_2025_31_MOESM2_ESM.pdf]

nature portfolio

Corresponding author(s): Hilary Brumberg

Last updated by author(s): Dec 9, 2025

Reporting Summary

Nature Portfolio wishes to improve the reproducibility of the work that we publish. This form provides structure for consistency and transparency in reporting. For further information on Nature Portfolio policies, see our Editorial Policies and the Editorial Policy Checklist.

Statistics

For all statistical analyses, confirm that the following items are present in the figure legend, table legend, main text, or Methods section.

n/a	Confirmed	
☐	☒	The exact sample size (*n*) for each experimental group/condition, given as a discrete number and unit of measurement
☐	☒	A statement on whether measurements were taken from distinct samples or whether the same sample was measured repeatedly
☐	☒	The statistical test(s) used AND whether they are one- or two-sided<br>*Only common tests should be described solely by name; describe more complex techniques in the Methods section.*
☐	☒	A description of all covariates tested
☐	☒	A description of any assumptions or corrections, such as tests of normality and adjustment for multiple comparisons
☐	☒	A full description of the statistical parameters including central tendency (e.g. means) or other basic estimates (e.g. regression coefficient) AND variation (e.g. standard deviation) or associated estimates of uncertainty (e.g. confidence intervals)
☐	☒	For null hypothesis testing, the test statistic (e.g. *F*, *t*, *r*) with confidence intervals, effect sizes, degrees of freedom and *P* value noted<br>*Give P values as exact values whenever suitable.*
☐	☒	For Bayesian analysis, information on the choice of priors and Markov chain Monte Carlo settings
☐	☒	For hierarchical and complex designs, identification of the appropriate level for tests and full reporting of outcomes
☐	☒	Estimates of effect sizes (e.g. Cohen's *d*, Pearson's *r*), indicating how they were calculated

Our web collection on statistics for biologists contains articles on many of the points above.

Software and code

Policy information about availability of computer code

Data collection	Original code was generated for data collection. Python 3.11.5, Google Earth Engine, ArcGISPro Version 3.1.4, and QGIS Version 3.36.2-Maidenhead were used for data extraction.
Data analysis	Original code was generated for data analysis. Data analysis was conducted in R-Studio Version 2024.04.1.

For manuscripts utilizing custom algorithms or software that are central to the research but not yet described in published literature, software must be made available to editors and reviewers. We strongly encourage code deposition in a community repository (e.g. GitHub). See the Nature Portfolio guidelines for submitting code & software for further information.

Data

Policy information about availability of data

All manuscripts must include a data availability statement. This statement should provide the following information, where applicable:

- Accession codes, unique identifiers, or web links for publicly available datasets
- A description of any restrictions on data availability
- For clinical datasets or third party data, please ensure that the statement adheres to our policy

Data used for the analysis and the land use and land cover maps have been deposited in Harvard Dataverse and will be made publicly-available upon publication.

Research involving human participants, their data, or biological material

Policy information about studies with human participants or human data. See also policy information about sex, gender (identity/presentation), and sexual orientation and race, ethnicity and racism.

Reporting on sex and gender	NA
Reporting on race, ethnicity, or other socially relevant groupings	NA
Population characteristics	NA
Recruitment	NA
Ethics oversight	NA

Note that full information on the approval of the study protocol must also be provided in the manuscript.

Field-specific reporting

Please select the one below that is the best fit for your research. If you are not sure, read the appropriate sections before making your selection.

☐ Life sciences ☐ Behavioural & social sciences ☒ Ecological, evolutionary & environmental sciences

For a reference copy of the document with all sections, see nature.com/documents/nr-reporting-summary-flat.pdf

Ecological, evolutionary & environmental sciences study design

All studies must disclose on these points even when the disclosure is negative.

Study description	We used a suite of causal inference models to help disentangle the impacts of land use on gulf turbidity at different spatial scales. There are many confounding factors that may affect any given land use in southern Costa Rica and turbidity in the Golfo Dulce, which we identified through a literature review and experience in the region. For each treatment land use, these confounders include other land uses (including roads), the proportion of that land use in other parts of the watershed, slope, soil type, and other unobserved confounders. To estimate causal impacts, we applied four complementary causal inference models—inverse probability of treatment weighting (IPTW), two-way fixed effects, group mean covariate, and group mean centered—alongside naïve and mixed-effects models, as a robustness check because each model makes different assumptions. IPTW was our primary approach because it best balanced covariates across treatment groups while retaining a flexible, interpretable framework suited for our spatially heterogeneous, observational dataset.
Research sample	Our sampling included 119 unique river mouths across 64 watersheds that flow directly into the Golfo Dulce. Each location was sampled three times: 1987, 1998, and 2019. At each of these locations for each year, we extracted gulf turbidity at 14 offshore distances (25-800m).
Sampling strategy	We generated a map of rivers that flow into the Golfo Dulce by compiling river layers from the Digital Atlas of Costa Rica 2014, the recent orthophoto and cartography initiative from the Costa Rican National Registry, and manually digitizing rivers based on topography and satellite imagery. We considered river mouths to be unique if they were at least 300 meters apart; consequently, some rivers have multiple river mouths.
Data collection	The main datasets used in this analysis were derived from freely available NASA satellite imagery, Costa Rican open-source datasets, and in situ data collected by collaborators. Data extraction was conducted in Google Earth Engine (GEE), Python, and QGIS, and data analysis and visualization were conducted in R.
Timing and spatial scale	We analyzed the effect of land use on gulf turbidity in three years: 1987, 1998, and 2019. These years were selected to illustrate changes in the region over a long period to be able to capture the impacts of policy-responsive land use changes on gulf turbidity and due to the lagging effects of land use changes on downstream turbidity. Those three years in particular were chosen because they satellite imagery with little cloud interference, which can be rare in the moist tropics (Brumberg et al., 2024). Land use and NDTI data are at 30m resolution, the resolution of the Landsat satellites. We quantified the effects of land use at two terrestrial spatial scales—entire watersheds and 15-meter-wide riparian zones—on gulf turbidity across 14 offshore distances (25-800m).
Data exclusions	Not applicable.
Reproducibility	We compared a suite of seven modelling approaches, each with different assumptions, to evaluate the robustness of our results. The data will be made open access upon publication. In the manuscript, we discuss how the methods could be replicated in other regions to evaluate the impact of land use on marine ecosystems in other areas.
Randomization	This is an observational dataset/natural experiment, so treatment is inherently non-random. Consequently, we used a suite of causal inference methods to help account for the non-random treatment in order to disentangle factors that confound with the treatment.

Blinding	This is not relevant because this research was not conducted on human subjects and it is an observational dataset/natural experiment.

Did the study involve field work? ☐ Yes ☒ No

Reporting for specific materials, systems and methods

We require information from authors about some types of materials, experimental systems and methods used in many studies. Here, indicate whether each material, system or method listed is relevant to your study. If you are not sure if a list item applies to your research, read the appropriate section before selecting a response.

Materials & experimental systems

n/a	Involved in the study
☒	☐ Antibodies
☒	☐ Eukaryotic cell lines
☒	☐ Palaeontology and archaeology
☒	☐ Animals and other organisms
☒	☐ Clinical data
☒	☐ Dual use research of concern
☒	☐ Plants

Methods

n/a	Involved in the study
☒	☐ ChIP-seq
☒	☐ Flow cytometry
☒	☐ MRI-based neuroimaging

Plants

Seed stocks	NA
Novel plant genotypes	NA
Authentication	NA
